# The effect of quercetin supplementation on clinical outcomes in COVID‐19 patients: A systematic review and meta‐analysis

**DOI:** 10.1002/fsn3.3715

**Published:** 2023-09-26

**Authors:** Somayeh Ziaei, Malek Alimohammadi‐Kamalabadi, Motahareh Hasani, Mahsa Malekahmadi, Emma Persad, Javad Heshmati

**Affiliations:** ^1^ ICU Department, Emam Reza Hospital Kermanshah University of Medical Sciences Kermanshah Iran; ^2^ Department of Cellular‐Molecular Nutrition, School of Nutritional Sciences and Dietetics Tehran University of Medical Sciences Tehran Iran; ^3^ Department of Nutritional Sciences, School of Health Golestan University of Medical Sciences Gorgan Iran; ^4^ Imam Khomeini Hospital Complex, Tehran University of Medicinal Sciences Tehran Iran Tehran University of Medical Sciences Tehran Iran; ^5^ Department for Evidence‐based Medicine and Evaluation Danube University Krems Krems Austria; ^6^ Songhor Healthcare Center Kermanshah University of Medical Sciences Kermanshah Iran

**Keywords:** COVID‐19, CRP, D‐dimmer, ferritin, LDH, mortality, quercetin

## Abstract

Coronavirus disease (COVID‐19) affects both the respiratory system and the body as a whole. Natural molecules, such as flavonoid quercetin, as potential treatment methods to help patients combat COVID‐19. The aim of this systematic review and meta‐analysis is to give a comprehensive overview of the impact of quercetin supplementation on inflammatory factors, hospital admission, and mortality of patients with COVID‐19. The search has been conducted on PubMed, Scopus, Web of Science, EMBASE, and the Cochrane Library using relevant keywords until August 25, 2023. We included randomized controlled trials (RCTs) comparing COVID‐19 patients who received quercetin supplementation versus controls. We included five studies summarizing the evidence in 544 patients. Meta‐analysis showed that quercetin administration significantly reduced LDH activity (standard mean difference (SMD): −0.42, 95% CI: −0.82, −0.02, *I* ^2^ = 48.86%), decreased the risk of hospital admission by 70% (RR: 0.30, 95% CI: 0.14, 0.62, *I* ^2^ = 00.00%), ICU admission by 73% (RR: 0.27, 95% CI: 0.09, 0.78, *I* ^2^ = 20.66%), and mortality by 82% (RR: 0.18, 95% CI: 0.03, 0.98, *I* ^2^ = 00.00%). No significant changes in CRP, D‐dimmer, and ferritin were found between groups. Quercetin was found to significantly reduce LDH levels and decrease the risk of hospital and ICU admission and mortality in patients with COVID‐19 infection.

## Graphical abstract

Our findings demonstrate that quercetin administration significantly reduced LDH activity, decreased the risk of hospital and ICU admission by 30% and 27%, respectively, and decreased mortality by 18% in patients with COVID‐19 infection. Our study provides a comprehensive overview of the effect of quercetin supplementation on clinical outcomes in COVID‐19 patients.

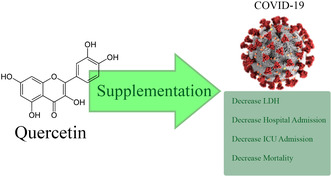

## 1 INTRODUCTION

COVID‐19 is an extremely contagious respiratory disease that is caused by a newly identified type of coronavirus, known as SARS‐CoV‐2 (Sullivan et al., 2020). The disease was first identified in Wuhan, China, in December 2019, and quickly spread to become a global pandemic (Pollard et al., 2020). To date, COVID‐19 has infected millions of people worldwide, resulting in a significant number of deaths (Noor et al., 2020). According to the WHO's website on March 7, 2023, there have been 759,408,703 confirmed cases of COVID‐19 and 6,866,434 confirmed deaths directly or due to consequences of this pandemic infection (Melnyk et al., 2023). COVID‐19 can cause mild symptoms, such as coughing, diarrhea, high fever, muscle pain, fatigue, and a significant decrease in the sense of smell (Nehme et al., 2021). However, if left untreated, it can lead to serious health complications. These may include acute respiratory distress syndrome, significant damage to the lungs, a high level of inflammatory cytokines, also known as cytokine storm, which can result in organ failure, pulmonary embolism or embolism in other organs, and even death (Cirulli et al., 2020; Seeßle et al., 2022). Several endeavors have been made within the medical industry to slow viral transmission and establish effective methods of treating individuals who test positive for SARS‐CoV‐2 (Memon et al., 2021; Morato et al., 2020).

In COVID‐19 pathophysiology, lactate dehydrogenase (LDH), C‐reactive protein (CRP), ferritin, and D‐dimer levels are commonly observed to be deregulated (Henry et al., 2020; Rostami & Mansouritorghabeh, 2020; Vargas‐Vargas & Cortés‐Rojo, 2020; Wang, 2020). CRP is an acute‐phase protein that increases during high inflammation situations. It has been observed that CRP levels significantly increase with COVID‐19 infection, indicating an immune response to the viral infection. (Kadkhoda, 2020). LDH is an enzyme released from damaged cells and is often used as a marker for tissue damage (Szarpak et al., 2021). In COVID‐19, LDH levels have been observed to be elevated, suggesting tissue damage caused by the virus (Fialek et al., 2022). Ferritin is a protein that stores iron in the body and is often used as a marker of inflammation (Banchini et al., 2021). In COVID‐19 patients, ferritin levels have been found to be significantly elevated, indicating a strong immune response to the viral infection (Faghih Dinevari et al., 2021). D‐dimer is a protein fragment that is produced when blood clots are broken down, and elevated levels of D‐dimer have been observed in COVID‐19 patients, suggesting a hypercoagulable state and an increased risk of thrombosis (Naymagon et al., 2020). These biomarkers can be useful in monitoring the progression of COVID‐19 and guiding treatment decisions.

Numerous scientific groups worldwide are currently investigating different natural molecules as potential treatments for COVID‐19 (Heidari et al., 2022; Heidary et al., 2020; Xiong et al., 2020). One of the active molecules is quercetin, a flavonoid that can be found in various natural sources such as fruits, legumes, herbs, vegetables, and bee products (Okamoto, 2005). Quercetin has also been found to have antiviral properties and has shown effectiveness in treating SARS and MERSS (Gasmi et al., 2022). Belonging to the flavonoid class of polyphenols, quercetin (3,3′,4′,5,7‐pentahydroxyflavone) is a significant component found in several food sources, including onion peels, nuts, fruits, wine, vegetables, and black tea (Kressler et al., 2011). A growing body of evidence suggests that the consumption of quercetin or quercetin‐rich foods can provide various health benefits, such as reducing blood clotting, inflammation, high blood pressure, and high blood sugar levels, and can have a positive impact on lipid metabolism disorders (Ostadmohammadi et al., 2019; Sahebkar, 2017; Serban et al., 2016). There are also several studies that provide evidence of the antiviral and antibacterial effects of quercetin (Chaabi, 2020; Sun et al., 2021; Wong et al., 2017).

This review aims to examine the impact of quercetin on COVID‐19 patients by analyzing its effects on CRP, LDH enzymes, ferritin, and d‐dimer levels, as well as its potential to reduce mortality rates, hospital stay, and ICU admissions.

## 2 METHODS

This study followed the preferred reporting items for systematic reviews and meta‐analysis (PRISMA) guidelines (Moher et al., 2009) and was prospectively registered in PROSPERO (CRD42023407390).

### 2.1 Design

Scopus, Medline, Web of Science, EMBASE, and Cochrane Library were utilized to search for studies using a combination of the search terms “COVID‐19” OR “SARS‐CoV‐2” OR “Coronavirus” OR “Coronavirus Disease 2019” OR “Novel Coronavirus, 2019” AND “Quercetin” OR “Quercetol” OR “Flavonol” OR “Dikvertin” OR “3,3′,4′,5,7‐Pentahydroxyflavone” OR “Sophoretin” OR “Meletin” OR “Xanthaurine” in titles and abstracts and in combination with MESH terms. The complete search terms and synthases are presented in File S1. The search was not limited by language and was focused on human studies. A search for unpublished academic papers, such as dissertations and theses, using the databases was also conducted. In addition, the reference lists of all the articles included in the study alongside any relevant narrative reviews to identify other relevant studies were examined. The literature search covered from inception to August 25, 2023.

### 2.2 Study selection

The criteria for inclusion of original studies in this meta‐analysis were as follows: (1) randomized clinical trials using either a parallel or crossover design with a placebo control group, (2) investigating the effects of quercetin on COVID‐19 patients, (3) sufficient information presented on baseline and final variables, namely CRP, LDH enzyme activity, ferritin, d‐dimmer, mortality, length of stay in hospital, and ICU admission, and (4) administration of quercetin for a minimum of 1 week. Studies were excluded if they were: (1) review, case reports, and letter to editor studies, (2) nonclinical, (3) uncontrolled trials, (4) lacking sufficient information on baseline or follow‐up of interested variables.

Two researchers conducted database searches and used the EndNote X7 version (Clarivate Analytics, Sydney, Australia) to remove duplicate records. Titles and abstracts of the remaining studies were dually screened based on the PICOS eligibility criteria. Following this, full‐text screening was performed by both reviewers. Any discrepancies were resolved through discussion with a third investigator.

### 2.3 Data extraction and quality assessment

In this systematic review, the following data were extracted: the name of the first author, the publication year, the place where the study was conducted, the number of participants, the time of supplementation and dose of quercetin, the age and gender of the participants, and the effects that were observed. Using the Cochrane criteria, risk of bias assessment of the included studies was conducted. The evaluation involved examining each study for factors, such as proper sequence generation, allocation concealment, blinding, management of incomplete outcome data, selective outcome reporting, and other sources of potential bias. To determine the level of bias, a “yes” judgment indicated a low risk of bias, while a “no” judgment indicated a high risk of bias. If an item could not be determined, it was labeled as “unclear” to signify an unknown or ambiguous risk of bias, following the guidelines of the Cochrane Handbook (Reeves, 2011).

### 2.4 Statistical analysis

The statistical analysis of the study was conducted using STATA 17 (Stata Corporation). The meta‐analyses of dichotomous outcomes, such as mortality, ICU admission, and hospitalization events, were performed using the Mantel–Haenszel method and a fixed effects model due to the low heterogeneity between the included trials. Results were presented as risk ratios with a 95% confidence interval. For continuous outcomes, such as CRP, LDH, ferritin, and D‐dimmer, due to higher heterogeneity, standard mean differences (SMD) including 95% confidence interval (CI) and random effect model (restricted maximum‐likelihood method) were used. Meta‐analyses were presented in forest plots. Due to the limited number of included studies, the only subgroup analysis performed was based on quercetin doses. The heterogeneity between studies included in one meta‐analysis was assessed using the χ^2^ and *I* ^2^ statistic. Sensitivity analyses were conducted to exclude outliers in case of considerable heterogeneity (*I* ^2^ > 70%).

## 3 RESULTS

### 3.1 Study selection

**FIGURE 1:**
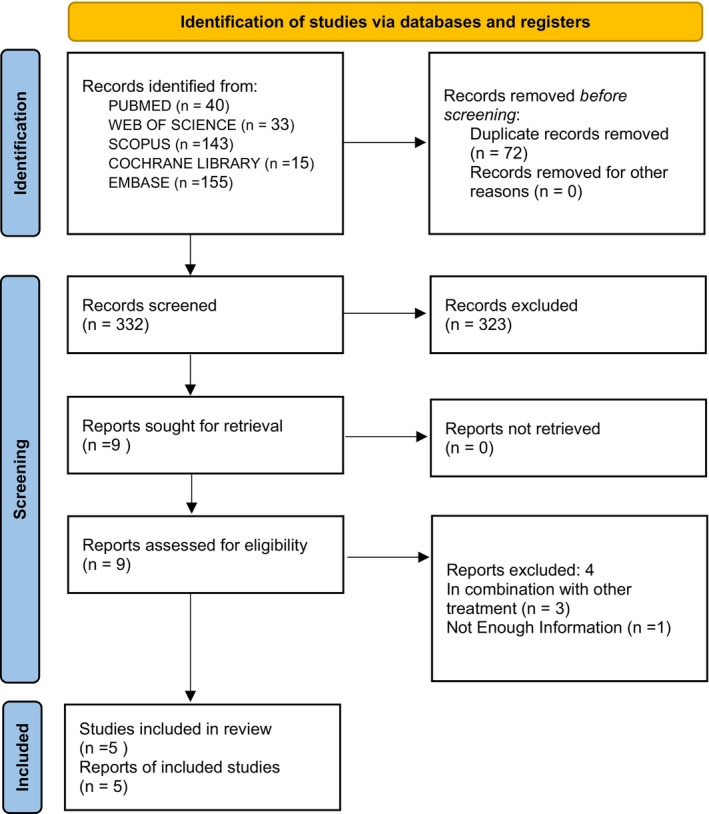
PRISMA Flow diagram of study selection.

Following deduplication, reviewers screened 332 articles, titles, and abstracts, excluding 323. The two reviewers then screened the nine full‐text articles, including five studies overall in the review (Di Pierro et al., 2023; Di Pierro, Derosa, et al., 2021; Di Pierro, Iqtadar, et al., 2021; Shohan et al., 2022; Zupanets et al., 2021). Figure 1 illustrates the selection process, along with the number of studies at each stage and the reasons for exclusion.

### 3.2 Study characteristics

**FIGURE 2:**
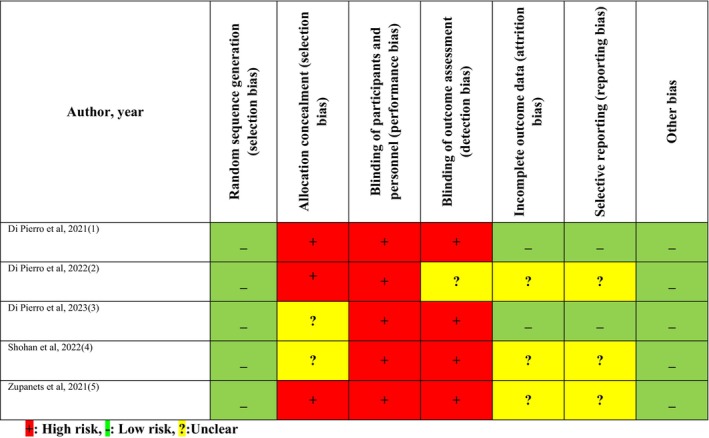
Assessment of the risk of bias in the included studies.

Table 1 presents the details of the included studies. Overall, 544 patients were enrolled across the five studies, with each individual study consisting of 42–200 participants The studies were conducted in Pakistan, Ukraine, and Iran and published between 2021 and 2023. All were double‐blinded randomized controlled trials. The mean age of participants ranged from 41.1 to 57.2 years and the dose of quercetin used varied from 480 to 1500 mg/day. The duration of quercetin supplementation varied between 1 and 4 weeks. Of the five studies, four provided information about the effect of quercetin on CRP and D‐dimmer. In addition, three studies reported the effect of quercetin on LDH and ferritin alongside hospitalization, ICU admission, and mortality. The results of the quality assessment of the included trials are presented in Figure 2, and the certainty of evidence for the meta‐analysis outcomes of each individual variable based on the GRADE approach is presented in File S2.

**TABLE 1:** Main characteristics of included studies.

Study (ref)	Country	Sample size	Quercetin dosage (mg/day)	Control type	Duration (week)	Gender (% females)	Age (years)		BMI (kg/m^2^)		Main outcome
							Intervention mean ± SD	Placebo mean ± SD	Intervention mean ± SD	Placebo mean ± SD	
Di Pierro, Iqtadar, et al. (2021)	Pakistan	42	600	Regular Covid Treatment	2	53	42.5 ± 3.3	56.2 ± 3.3			↓LDH, ↓Ferritin, ↓CRP, and ↓D‐dimer
Di Pierro, Derosa, et al. (2021)	Pakistan	152	1000	Regular Covid Treatment	4	42					↓length of stay, ↓ non‐invasive oxygen therapy, ↓ICU, and ↓mortality
Di Pierro et al. (2023)	Pakistan	100	1500	Regular Covid Treatment	2	52	41.1 ± 2.03	54.1 ± 2.03			↓LDH, ↔Ferritin, ↔CRP and ↔D‐dimer
Shohan et al. (2022)	Iran	60	1000	Regular Covid Treatment	1	43					↓ALP, ↓CRP, and ↓LDH
Zupanets et al. (2021)	Ukraine	200	480	Regular Covid Treatment	2	51	57.2 ± 12.06	54.3 ± 11.75	27,80 ± 4,62	27,10 ± 4,09	↔Ferritin, ↔CRP and ↓D‐dimer

*Note*: Symbol is a sign of decreasing variables in the intervention group, ↑ This symbol is a sign of increasing variables in the intervention group, ↔ This sign indicates that there is no difference between the two groups.

Abbreviations: ALP, Alkaline Phosphatase; CRP, C‐reactive protein; ICU, Intensive Care Unit; LDH, Lactate dehydrogenase; NR, not reported.

### 3.3 Meta‐analysis

**FIGURE 3:**
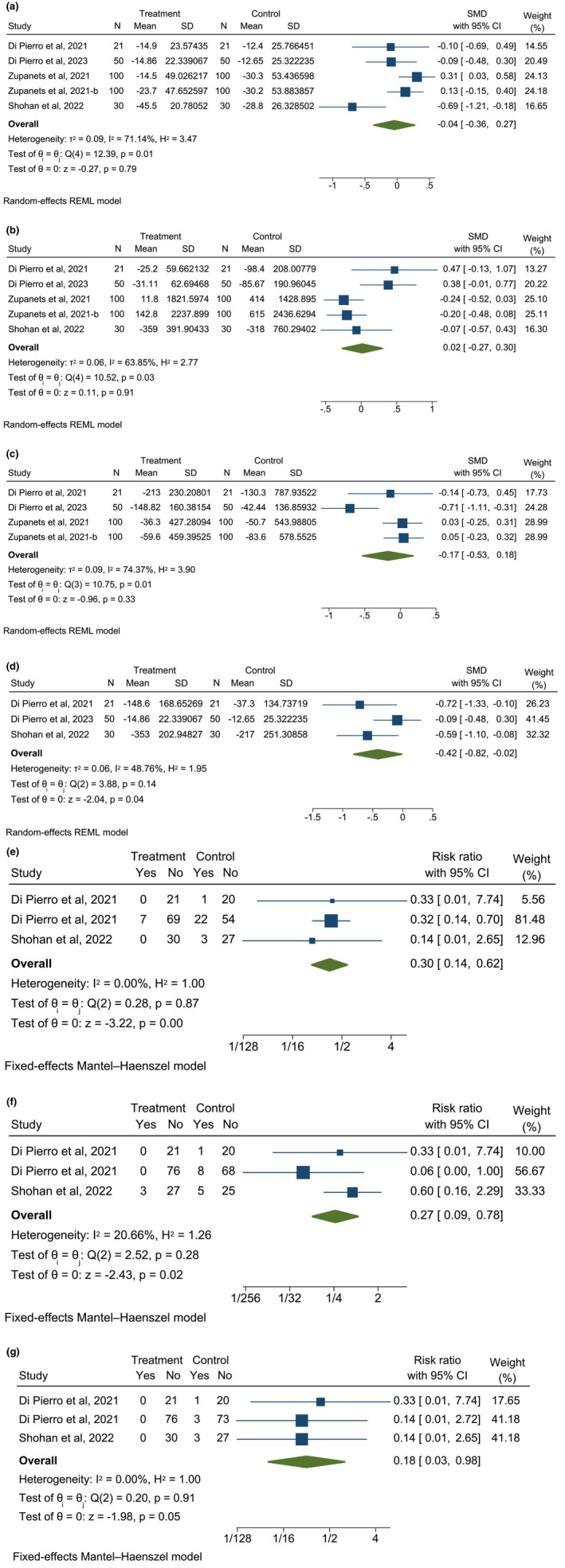
The effect of quercetin on CRP (a), D‐Dimmer (b), ferritin (c), LDH (d), risk of hospitalization (e), risk of ICU admission (f), and mortality (g) in COVID‐19 patients compared to standard treatment.

The meta‐analysis of data from three treatment arms showed significant reductions in LDH activity (SMD: −0.42, 95% CI: −0.82, −0.02, *I* ^2^ = 48.86%) following supplementation with quercetin (Figure 3d). Supplementation also reduced the risk of hospitalization by 70% (RR: 0.30, 95% CI: 0.14, 0.62, *I* ^2^ = 00.00%), risk of admission to the ICU by 73% (RR: 0.27, 95% CI: 0.09, 0.78, *I* ^2^ = 20.66%), and mortality by 82% (RR: 0.18, 95% CI: 0.03, 0.98, *I* ^2^ = 00.00%). The GRADE evaluation of the certainty of evidence, however, indicates that there is low certainty of evidence for this finding regarding LDH, hospitalization, ICU admission, and mortality. In addition, our meta‐analysis indicated that quercetin intake did not significantly change CRP (SMD: −0.04, 95% CI: −0.36, 0.27, *I* ^2^ = 71.14% *n* = 5), D‐dimmer (SMD: 0.02, 95% CI: −0.27, 0.30, *I* ^2^ = 63.85% *n* = 5), or ferritin (SMD: −0.17, 95% CI: −0.53, 0.18, *I* ^2^ = 74.37% *n* = 4) compared to standard treatment in COVID‐19 patients (Figure 3). However, these findings have a very low certainty of evidence, so they should be declared with caution. We performed sensitivity and subgroup analysis for continuous outcomes concerning CRP, LDH, ferritin, and D‐dimer due to higher heterogeneity. During sensitivity analysis, we dropped one study at a time, which showed that there was no significant change in the effect size after removing each individual study (File S3). Subgroup analysis based on quercetin dose also did not show any significant change in the pooled effect sizes (File S4).

## 4 DISCUSSION

To the best of our knowledge, this is the first systematic review and meta‐analysis conducted to investigate the effectiveness of quercetin in COVID‐19 patients, by analyzing enzyme activity and inflammatory markers. The main findings of this systematic review and meta‐analysis of RCTs indicate that quercetin supplementation may reduce LDH activity, the risk of hospitalization, ICU admission, and mortality in individuals with COVID‐19 infection. However, the results did not reveal any significant differences between the quercetin and the control groups regarding CRP, ferritin, and D‐dimer levels. Cheema et al. in 2023 found that although quercetin did not reduce the risk of mortality or promote recovery, it did lower the rate of hospital and ICU admission (Cheema et al., 2023), which is in line with our findings regarding hospital and ICU admission.

Several factors are associated with the morbidity and mortality of COVID‐19 patients, including male gender, obesity, cardiovascular diseases, cancer, smoking, and hyper‐response of immune system (de Lucena et al., 2020). The disproportionate reaction of the immune system has been linked to the pathogenesis of COVID‐19 (Darif et al., 2021). It has been observed that patients in the advanced stages of COVID‐19 experience uncontrolled systemic inflammatory reactions characterized by cytokine release syndrome (CRS), which is also seen in SARS and MERS patients (Del Valle et al., 2020; Moore & June, 2020).

The substantial release of proinflammatory cytokines by both immune and nonimmune cells is responsible for CRS. This, in turn, can cause a high level of inflammation in the lungs, leading to vast pulmonary damage (Hirano & Murakami, 2020). Furthermore, the excessive release of proinflammatory cytokines can lead to hyper‐coagulation and thrombosis, which can be fatal for individuals severely infected with SARS‐CoV‐2 (Gonzalez‐Rubio et al., 2020). CRP, LDH, D‐dimer, and ferritin have been used to evaluate the prognosis of the inflammatory status of COVID‐19 patients (Huang et al., 2020). Although our systematic review found no significant change in CRP, D‐dimer, and ferritin levels after quercetin administration, we found that quercetin could decrease the levels of LDH in COVID‐19 patients.

LDH, which is a cellular enzyme responsible for anaerobic glycolysis and facilitates the conversion of pyruvate to lactate, is commonly assessed in clinical settings to determine the presence of certain diseases (Szarpak et al., 2021). Increased concentrations of serum LDH have been associated with unfavorable outcomes in a range of ailments, particularly tumors and inflammatory conditions (Poggiali et al., 2020). It has been demonstrated so far that individuals with severe COVID‐19 exhibit elevated concentrations of LDH in their blood serum (Bartziokas & Kostikas, 2021). In the context of COVID‐19, elevated LDH levels have been associated with more severe disease and lung injury (Henry et al., 2020). It serves as a biomarker for assessing the extent of tissue damage and can help clinicians monitor disease progression and response to treatment (Santos et al., 2020). High LDH levels indicate potential organ damage, including lung damage, which can be clinically significant in guiding treatment decisions and predicting patient outcomes in COVID‐19 cases (Robilotti et al., 2020). It has been shown that high concentrations of LDH are associated with an approximately six‐fold increase in the likelihood of severe COVID‐19 complications and a 16‐fold increase in the likelihood of mortality among these patients (Henry et al., 2020).

Recently, there has been some evidence indicating that quercetin exhibits anti‐coronavirus properties, mostly through its anti‐inflammatory effects (Valério et al., 2009). To enter human cells, the COVID‐19 virus, being an RNA‐based virus, recruits the angiotensin‐converting enzyme 2 receptors (hACE‐2) (Gollapalli et al., 2022). The COVID‐19 virus uses hACE‐2 to initiate cascades that lead to high levels of proinflammatory cytokines, reactive oxygen species, and eventually acute respiratory distress syndrome (Kuppusamy et al., 2021). The replication of the COVID‐19 virus requires two key enzymes: a highly active protease named 3CLPro and RNA‐dependent RNA polymerase (RdRp). Primary studies using quercetin as an adjunct antioxidant therapy for COVID‐19 patients have shown that it can significantly inhibit the expression of hACE‐2 genes, which decreases the virus's ability to enter human cells. Additionally, it has been reported that quercetin treatment can suppress the activity of the two key enzymes involved in COVID‐19 replication, 3CLPro, and RdRp, ultimately leading to a decrease in the concentration of proinflammatory cytokines (Imran et al., 2022). Quercetin also displays other ways of reducing inflammation, such as stopping lipid‐peroxidation (Wagner et al., 2006), phospholipase A2 (Lättig et al., 2007), and lipoxygenase (Kim et al., 2016), and targeting the nucleotide‐binding oligomerization domain leucine‐rich repeat and pyrine domain‐containing protein 3 (NLRP3) to decrease the production of IL‐1ß (Li et al., 2021). Additionally, quercetin was found to decrease the concentration of reactive oxygen species produced during COVID‐19 infection. It also reduced the levels of nitric oxide induced by lipopolysaccharide (LPS). These findings suggest that quercetin possesses strong antioxidant and anti‐inflammatory properties (Lekić et al., 2013). In primary studies, it has been reported that quercetin improves the ability of antioxidant defense against oxidative stress by inducing the gene expression of active antioxidant enzymes, including superoxide dismutase, catalase, and glutathione peroxidase. The expression of these enzymes that quercetin stimulated also safeguards tissues from harm caused by oxidative damage (Granado‐Serrano et al., 2012).

Flavonoids, such as quercetin, possess various biological activities, such as acting as a potential antioxidant, antiviral, anti‐inflammatory, and antiallergic agent, as well as reducing damage to the liver. A significant characteristic of severe viral infections such as SARS and COVID‐19 is a cytokine storm, which involves the release of major inflammatory factors like interleukins, interferon‐gamma (IFN‐gamma), tumor necrosis factor‐α (TNF‐α), and other cytokines (Fara et al., 2020). Quercetin has strong immunomodulatory effects, as it can hinder the gene expression of a wide range of factors that affect the signaling pathways involved in the production of proinflammatory cytokines (Manjunath & Thimmulappa, 2022). It has been revealed that the use of quercetin can decrease the level of inflammation in airways by reducing the gene expression of various interleukins, such as IL‐5 and IL‐4. Additionally, quercetin was found to reduce the factors responsible for activating the NF‐κB pathway and the DNA gene expression of P‐selectins (Rogerio et al., 2010).

Our results found that quercetin may help control COVID‐19 by reducing hospitalization and ICU admissions and lowering mortality. Alongside the anti‐inflammatory and antioxidant effects of quercetin, its antiviral effect of quercetin should also be emphasized. The 3CLpro, which is also known as the main protease, and papain‐like protease (PLpro) are appealing targets for drug development because they are highly preserved in human coronaviruses (Roe et al., 2021). In COVID‐19 infection, the enzymes 3CLpro and PLpro are up‐regulated. These enzymes are responsible for separating specific structural proteins, including pp1a and pp1ab, into nonstructural proteins. These nonstructural proteins, such as RNA‐dependent RNA polymerase, are critical for the replication of the COVID‐19 virus (Anand et al., 2003). Recent research, both computational and experimental, has demonstrated that quercetin can restrain the activity of COVID‐19 3CLpro (Abian et al., 2020; Jo et al., 2019; Nguyen et al., 2012).

Furthermore, based on molecular docking studies, it has been found that quercetin has the ability to interact with one of the significant and active residues of the ACE2 receptor (Zhou et al., 2020). Interaction of quercetin with ACE2 residue could disrupt the affinity of the COVID‐19 virus to the host cell membrane (Bhowmik et al., 2021). Consequently, quercetin might be able to prevent COVID‐19 virus infection by impeding its entry into pulmonary cells through modifying the viral spike protein or host ACE2. The effect of quercetin was also observed in studies that administered quercetin in combination with other antioxidants. This suggests that quercetin may be the specific factor capable of decreasing ACE2 receptor activity in COVID‐19 patients (Onal et al., 2021).

Another potential mechanism explaining the positive effects of quercetin in COVID‐19 patients could be its impact on cholesterol and macrophage metabolism (Pawar et al., 2021). The interaction between cholesterol, macrophages, and SARS‐CoV‐2 is a critical focus of research. Lipids, such as cholesterol, play a fundamental role in cellular and viral membranes, thereby influencing viral replication (Gowdy & Fessler, 2013). SARS‐CoV‐2 infection triggers the upregulation of genes involved in lipogenesis and cholesterol synthesis, which are essential for the formation of viral membranes (Ehrlich et al., 2020). However, this heightened lipogenesis can lead to pulmonary lipotoxicity in delicate epithelial tissues. Elevated levels of cholesterol in the lungs can stimulate inflammation and lung diseases (Yan et al., 2019). Quercetin holds promise as it can modulate cholesterol metabolism by regulating the expression of ABCA1, a key regulator of reverse cholesterol transport, and it may also reduce the accumulation of cholesterol in macrophages (Zhang et al., 2016). It is believed that the positive effect of quercetin on COVID‐19 can be enhanced when it is accompanied by other nutritional and pharmaceutical factors (Pawar et al., 2022). It has been demonstrated that fenofibrate (Pawar et al., 2021) and dexamethasone (Pawar & Pal, 2020) synergistically enhance the effects of quercetin in improving respiratory and inflammatory complications in COVID‐19 patients, respectively.

Our systematic review and meta‐analysis had several limitations. First, we only included trials with small sample sizes, which resulted in imprecise outcomes that need to be verified by large‐scale RCTs. Second, due to the limited number of included studies, we could not perform subgroup analyses to determine the impact of various baseline variables, such as comorbidities, age, dose of the quercetin, early or late treatment, and severity of the COVID‐19. Third, the included articles administered quercetin in different formulations, which created a high heterogeneity between studies. Additionally, differences in the type of control group regimen used in the included studies may also limit the interpretation of current results. Finally, the generalizability of these results may be limited due to the fact that most of the trials were performed in Asian countries and had patients with various COVID‐19 severity levels.

## 5 CONCLUSION

The findings from this systematic review and meta‐analysis demonstrate its potential as a therapeutic agent against COVID‐19. We found that quercetin significantly reduces LDH levels and decreases the risk of hospitalization, ICU admission, and mortality in COVID‐19 patients. These beneficial effects are potentially attributable to its antioxidant, anti‐inflammatory, and immunomodulatory activities. However, the effects of quercetin on other important indicators in COVID‐19 patients, such as CRP, ferritin, and D‐dimer in particular, need to be evaluated in large, long‐duration RCTs. Due to the low certainty of evidence, these results should be interpreted with caution.

## AUTHOR CONTRIBUTIONS

**Somayeh Ziaei:** Conceptualization (equal); data curation (equal); supervision (equal); writing – original draft (equal); writing – review and editing (equal). **Malek Alimohammadi‐Kamalabadi:** Investigation (equal); methodology (equal); writing – original draft (equal); writing – review and editing (equal). **Motahareh Hasani:** Software (equal); writing – original draft (equal); writing – review and editing (equal). **Mahsa Malekahmadi:** Investigation (equal); writing – original draft (equal). **Emma Persad:** Writing – original draft (equal); writing – review and editing (equal). **Javad Heshmati:** Conceptualization (equal); data curation (equal); supervision (equal); writing – original draft (equal); writing – review and editing (equal).

## FUNDING INFORMATION

None.

## CONFLICT OF INTEREST STATEMENT

The authors have no competing interests to declare.

## CONSENT

All authors consent to publish this article in this journal.

## Supporting information


File S1.

File S2.

File S3.

File S4.

## Data Availability

Not Applicable.
