# Supplementary material for: The effect of quercetin supplementation on clinical outcomes in COVID‐19 patients: A systematic review and meta‐analysis
Source: Food Sci Nutr. 2023 Sep 26;11(12):7504–14. doi: 10.1002/fsn3.3715 (PMC10724618; doi:10.1002/fsn3.3715)
Supplement: Supplementary file 1 — File S1. [file FSN3-11-7504-s004.doc]

**Supplementary file 1**

**A systematic review and meta-analysis effect of quercetin on inflammatory markers and mortality in COVID-19 patients.**

| **Groups** | **Descriptors** |
| --- | --- |
| Outcome | “COVID-19” OR “SARS-CoV-2” OR “Coronavirus” OR “Coronavirus Disease 2019” OR “Novel Coronavirus, 2019” |
| Exposure | “Quercetin” OR “Quercetol” OR “Flavonol” OR “Dikvertin” OR “3,3',4',5,7-Pentahydroxyflavone” OR “Sophoretin” OR “Meletin” OR “Xanthaurine” |
| Setting | Randomized controlled trial OR controlled clinical trial OR randomized controlled trials OR random allocation OR double blind method OR single blind method OR clinical trial OR clinical trials OR placebos OR placebo OR random |

**PUBMED**

**Number of localized studies:** 40

**Limits:** -

**Number of studies after applying limits:** 40

|  | **Descriptors** | Number of studies reached |
| --- | --- | --- |
| **#1** | "covid 19"[All Fields] OR "covid 19"[MeSH Terms] OR "covid 19 vaccines"[All Fields] OR "covid 19 vaccines"[MeSH Terms] OR "covid 19 serotherapy"[All Fields] OR "covid 19 nucleic acid testing"[All Fields] OR "covid 19 nucleic acid testing"[MeSH Terms] OR "covid 19 serological testing"[All Fields] OR "covid 19 serological testing"[MeSH Terms] OR "covid 19 testing"[All Fields] OR "covid 19 testing"[MeSH Terms] OR "sars cov 2"[All Fields] OR "sars cov 2"[MeSH Terms] OR "severe acute respiratory syndrome coronavirus 2"[All Fields] OR "ncov"[All Fields] OR "2019 ncov"[All Fields] OR (("coronavirus"[MeSH Terms] OR "coronavirus"[All Fields] OR "cov"[All Fields]) AND 2019/11/01:3000/12/31[Date - Publication]) OR ("coronavirus"[MeSH Terms] OR "coronavirus"[All Fields] OR "coronaviruses"[All Fields]) OR ("sars cov 2"[MeSH Terms] OR "sars cov 2"[All Fields] OR "sars cov 2"[All Fields]) OR ("covid 19"[MeSH Terms] OR "covid 19"[All Fields] OR "coronavirus disease 2019"[All Fields]) OR ("sars cov 2"[MeSH Terms] OR "sars cov 2"[All Fields] OR "novel coronavirus 2019"[All Fields]) | 360476 |
| **#2** | "quercetin"[MeSH Terms] OR "quercetin"[All Fields] OR "quercetin s"[All Fields] OR "quercetine"[All Fields] OR "quercetins"[All Fields] OR "Quercetol"[All Fields] OR ("3 hydroxyflavone"[Supplementary Concept] OR "3 hydroxyflavone"[All Fields] OR "flavonol"[All Fields] OR "flavonols"[MeSH Terms] OR "flavonols"[All Fields]) OR ("quercetin"[MeSH Terms] OR "quercetin"[All Fields] OR "dikvertin"[All Fields]) OR ("quercetin"[MeSH Terms] OR "quercetin"[All Fields] OR ("3 3 4 5 7"[All Fields] AND "pentahydroxyflavone"[All Fields]) OR "3 3 4 5 7 pentahydroxyflavone"[All Fields]) OR ("quercetin"[MeSH Terms] OR "quercetin"[All Fields] OR "sophoretin"[All Fields]) OR "Meletin"[All Fields] OR "miletin"[All Fields] OR "Xanthaurine"[All Fields] | 36179 |
| **#3** | ((((((((("Randomized Controlled Trial"[Publication Type] OR "Controlled Clinical Trial"[Publication Type]) OR "Randomized Controlled Trials as Topic"[Mesh]) OR "Random Allocation"[Mesh]) OR "Double-Blind Method"[Mesh]) OR "Single-Blind Method"[Mesh]) OR "Clinical Trial"[Publication Type]) OR ("clinical trial"[Publication Type] OR "clinical trials as topic"[MeSH Terms] OR "clinical trials"[All Fields])) OR "Placebos"[Mesh]) OR ("placebos"[MeSH Terms] OR "placebos"[All Fields] OR "placebo"[All Fields])) OR ("random allocation"[MeSH Terms] OR ("random"[All Fields] AND "allocation"[All Fields]) OR "random allocation"[All Fields] OR "random"[All Fields]) | 1953659 |
| **#4** | **#1** AND **#2** AND #3 | 40 |

**SCOPUS**

**Number of localized studies:** 143

**Limits:** *-*

**Number of studies after applying limits:** 143

|  | **Descriptors** | Number of studies reached |
| --- | --- | --- |
| **#1** | ( TITLE-ABS-KEY ( covid-19 ) ) OR ( TITLE-ABS-KEY ( sars-cov-2 ) ) OR ( TITLE-ABS-KEY ( coronavirus ) ) OR ( TITLE-ABS-KEY ( coronavirus AND disease 2019 ) ) OR ( TITLE-ABS-KEY ( novel AND coronavirus, 2019 ) ) | 504264 |
| **#2** | ( TITLE-ABS-KEY ( quercetin ) ) OR ( TITLE-ABS-KEY ( quercetol ) ) OR ( TITLE-ABS-KEY ( flavonol ) ) OR ( TITLE-ABS-KEY ( dikvertin ) ) OR ( TITLE-ABS-KEY ( 3,3',4',5,7-pentahydroxyflavone ) ) OR ( TITLE-ABS-KEY ( sophoretin ) ) OR ( TITLE-ABS-KEY ( meletin ) ) OR ( TITLE-ABS-KEY ( xanthaurine ) ) | 70150 |
| **#3** | ( TITLE-ABS-KEY ( randomized AND controlled AND trial ) OR TITLE-ABS-KEY ( controlled AND clinical AND trial ) OR TITLE-ABS-KEY ( randomized AND controlled AND trials ) OR TITLE-ABS-KEY ( random AND allocation ) OR TITLE-ABS-KEY ( double AND blind AND method ) OR TITLE-ABS-KEY ( single AND blind AND method ) OR TITLE-ABS-KEY ( clinical AND trial ) OR TITLE-ABS-KEY ( clinical AND trials ) OR TITLE-ABS-KEY ( placebos ) OR TITLE-ABS-KEY ( placebo ) OR TITLE-ABS-KEY ( random ) ) | 3714073 |
| **#4** | **#1** AND **#2** AND **#3** | 143 |

**COCHRANE**

**Number of localized studies: 15**

**Limits:** -

**Number of studies after applying limits:** 15

|  | **Descriptors** | Number of studies reached |
| --- | --- | --- |
| **#1** | Me ("COVID-19") or ("SARS-CoV-2"):ti,ab,kw or ("SARS-CoV-2"):ti,ab,kw or ("Coronavirus") or ("Coronavirus Disease 2019"):ti,ab,kw or ("Novel Coronavirus, 2019"):ti,ab,kw | 9356 |
| **#2** | Me ("Quercetin") or ("Quercetol"):ti,ab,kw or ("Flavonol"):ti,ab,kw or ("Dikvertin") or ("3,3',4',5,7-Pentahydroxyflavone"):ti,ab,kw or ("Sophoretin"):ti,ab,kw or ("Meletin"):ti,ab,kw or ("Xanthaurine"):ti,ab,kw | 88 |
| **#3** | **#1** AND **#2** | 15 |

**WEB OF SCIENCE**

**Number of localized studies:** 65

**Limits:** documents types (articles)

**Number of studies after applying limits:** 33

|  | **Descriptors** | Number of studies reached |
| --- | --- | --- |
| **#1** | TS=(“COVID-19”) OR TS=(“SARS-CoV-2”) OR TS=(“Coronavirus”) OR TS=(“Coronavirus Disease 2019”) OR TS=(“Novel Coronavirus, 2019”) | 473218 |
| **#2** | TS=(“Quercetin”) OR TS=(“Quercetol”) OR TS=(“Flavonol”) OR TS=(“Dikvertin”) OR TS=(“3,3',4',5,7-Pentahydroxyflavone”) OR TS=(“Sophoretin”) OR TS=(“Meletin”) OR TS=(“Xanthaurine”) | 42177 |
| **#3** | TS=(Randomized controlled trial) OR TS=(controlled clinical trial) OR TS=(randomized controlled trials) OR TS=(random allocation) OR TS=(double blind method) OR TS=(single blind method) OR TS=(clinical trial) OR TS=(clinical trials) OR TS=(placebos) OR TS=(placebo) OR TS=(random) | 1689096 |
| **#4** | **#1** AND **#2** AND **#3** | 65 |

**EMBASE**

**Number of localized studies:** 155

**Limits:** no limits

|  | **Descriptors** | Number of studies reached |
| --- | --- | --- |
| **#1** | quercetin OR quercetol OR flavonol OR dikvertin OR pentahydroxyflavone OR sophoretin OR meletin OR xanthaurine | 58314 |
| **#2** | 'covid 19'/exp OR 'covid 19' OR 'sars cov 2' OR coronavirus OR (coronavirus AND disease AND 2019) OR (novel AND coronavirus, AND 2019) | 467139 |
| **#3** | (randomized AND controlled AND ('trial'/exp OR trial)) OR (controlled AND ('clinical'/exp OR clinical) AND ('trial'/exp OR trial)) OR (randomized AND controlled AND trials) OR (random AND allocation) OR (double AND ('blind'/exp OR blind) AND ('method'/exp OR method)) OR (single AND ('blind'/exp OR blind) AND ('method'/exp OR method)) OR (('clinical'/exp OR clinical) AND ('trial'/exp OR trial)) OR (('clinical'/exp OR clinical) AND trials) OR ('placebos'/exp OR placebos) OR ('placebo'/exp OR placebo) OR (random) | 3367529 |
| **#4** | **#1** AND **#2** AND **#3** | 155 |
