# Supplementary material for: The effect of quercetin supplementation on clinical outcomes in COVID‐19 patients: A systematic review and meta‐analysis
Source: Food Sci Nutr. 2023 Sep 26;11(12):7504–14. doi: 10.1002/fsn3.3715 (PMC10724618; doi:10.1002/fsn3.3715)
Supplement: Supplementary file 2 — File S2. [file FSN3-11-7504-s003.docx]

**Question:** Quercetin compared to Routine treatment for COVID-19

**Setting:**

**Bibliography:**

| **Certainty assessment** | | | | | | | **№ of patients** | | **Effect** | | **Certainty** | **Importance** |
| --- | --- | --- | --- | --- | --- | --- | --- | --- | --- | --- | --- | --- |
| **№ of studies** | **Study design** | **Risk of bias** | **Inconsistency** | **Indirectness** | **Imprecision** | **Other considerations** | **Quercetin** | **Routine treatment** | **Relative (95% CI)** | **Absolute (95% CI)** |  |  |
| **CRP** | | | | | | | | | | | | |
| 5 | randomised trials | serious^a^ | serious^b^ | serious^c^ | serious^d^ | none |  |  | - | **0**  (0 to 0 ) | ⨁◯◯◯ Very low |  |
| **D-Dimmer** | | | | | | | | | | | | |
| 5 | randomised trials | serious^a^ | serious^b^ | serious^c^ | serious^d^ | none |  |  | - | **0**  (0 to 0 ) | ⨁◯◯◯ Very low |  |
| **Ferritin** | | | | | | | | | | | | |
| 4 | randomised trials | serious^a^ | serious^b^ | serious^c^ | serious^d^ | none |  |  | - | **0**  (0 to 0 ) | ⨁◯◯◯ Very low |  |
| **LDH** | | | | | | | | | | | | |
| 3 | randomised trials | serious^a^ | not serious | serious^c^ | not serious | none |  |  | - | **0**  (0 to 0 ) | ⨁⨁◯◯ Low |  |
| **Hospitalization** | | | | | | | | | | | | |
| 3 | randomised trials | serious^a^ | not serious | serious^c^ | not serious | none |  |  | not estimable |  | ⨁⨁◯◯ Low |  |
| **ICU admission** | | | | | | | | | | | | |
| 3 | randomised trials | serious^a^ | not serious | serious^c^ | not serious | none |  |  | not estimable |  | ⨁⨁◯◯ Low |  |
| **Mortality** | | | | | | | | | | | | |
| 3 | randomised trials | serious^a^ | not serious | serious^c^ | not serious | none |  |  | not estimable |  | ⨁⨁◯◯ Low |  |

**CI:** confidence interval

#### Explanations

a. Serious risk of bias since more than 50 % of participants were from high-risk bias studies. Downgraded

b. Serious Inconsistency since I2> 60%. Downgraded

c. Serious Indirectness since more than 50% of participants come from a specific community. Downgraded

d. Serious Impression since the result of the meta-analysis was not statistically significant. Downgraded

**Supplementary File 2.** GRADE; certainty of evidence for the effect of quercetin on COVID-19 patients.
