# Supplementary material for: The effect of quercetin supplementation on clinical outcomes in COVID‐19 patients: A systematic review and meta‐analysis
Source: Food Sci Nutr. 2023 Sep 26;11(12):7504–14. doi: 10.1002/fsn3.3715 (PMC10724618; doi:10.1002/fsn3.3715)
Supplement: Supplementary file 3 — File S3. [file FSN3-11-7504-s002.docx]

A

B

C

D

**Supplementary File 3.** Sensetivity analysis of the effect of quercetin on CRP (A), D-Dimmer(B), Ferritin(C), and LDH(D) in COVID-19 patients compare to standard treatment.
