# Supplementary material for: The effect of quercetin supplementation on clinical outcomes in COVID‐19 patients: A systematic review and meta‐analysis
Source: Food Sci Nutr. 2023 Sep 26;11(12):7504–14. doi: 10.1002/fsn3.3715 (PMC10724618; doi:10.1002/fsn3.3715)
Supplement: Supplementary file 4 — File S4. [file FSN3-11-7504-s001.docx]

A

B

**Supplementary File 4.** Subgroup analysis based on quercetin dosage on CRP (A), and D-Dimmer (B) in COVID-19 patients compare to standard treatment.
